# Live Attenuated Bacterial Vectors as Vehicles for DNA Vaccine Delivery: A Mini Review

**DOI:** 10.21315/mjms2024.31.6.2

**Published:** 2024-12-31

**Authors:** Sze Wei Eng, Vilassini Muniandy, Lohshinni Punniamoorthy, Hui Xian Tew, Mohd Nor Norazmi, Manickam Ravichandran, Su Yin Lee

**Affiliations:** 1 Faculty of Applied Sciences, AIMST University, Kedah, Malaysia; 2 Centre of Excellence for Vaccine Development (CoEVD), Faculty of Applied Science, AIMST University, Kedah, Malaysia; 3 School of Health Sciences, Universiti Sains Malaysia, Kelantan, Malaysia; 4 Malaysia Genome and Vaccine Institute, National Institutes of Biotechnology Malaysia, Selangor, Malaysia; 5 MyGenome Sdn Bhd, Kuala Lumpur, Malaysia

**Keywords:** DNA, vaccine, live attenuated bacteria, delivery vehicles

## Abstract

DNA vaccines are third-generation vaccines composed of plasmids that encode vaccine antigens. Their advantages include fast development, safety, stability, and cost effectiveness, which make them an attractive vaccine platform for genetic and infectious diseases. However, the low transfection efficiency of DNA vaccines results in poor performance in both larger animals and humans, thereby limiting their clinical use. To overcome this issue, live attenuated bacterial vector (LABV) has been proposed as a DNA delivery vehicle. LABV is known to improve DNA vaccine transfection efficiency, thus enhancing the immune response. This article highlights recent advancements in the development of LABV DNA vaccines, the design of shuttle plasmids and adjuvants, and the potential applications of LABV candidates.

## Introduction

Infectious diseases cause major health crises that lead to the breakdown of the healthcare system, especially in developing and underdeveloped countries. Over the past few decades, the emergence of multidrug resistance (MDR) pathogenic bacteria, such as *Staphylococcus aureus* and *Mycobacterium tuberculosis*, has led to difficulties in treating several diseases, consequently increasing the clinical burden (1). Furthermore, in the past three years, the COVID-19 pandemic has had severe negative impacts on healthcare and economic systems worldwide. In this context, vaccination is one of the most effective approaches to control and halt the spread of MDR bacteria and viruses.

Conventional vaccines, such as killed or live attenuated vaccines, are efficient in eliciting antigen-specific antibodies to block the entry of pathogens into the host. Unfortunately, the rapid emergence of MDR bacteria strains and new antigenic mutants of infectious viruses has led to the loss of the protective efficacy of immunised people (2). Genetic vaccine, a third-generation vaccine platform, is a potent method for counteracting these rapidly mutating bacteria and viruses. Genetic vaccines are circular plasmids containing gene encoding target antigens that can be administered through non-parenteral routes (3). Notably, they are also termed DNA vaccines, RNA vaccines, and plasmid vaccines. This platform offers more advantages compared to conventional vaccine platforms, including specific immune cell targeting, multiple antigens vaccine construct, and rapid production (4).

Since mucosal surfaces, such as nasal, oral, and vaginal surfaces, are common routes used by pathogens to invade hosts, mucosal vaccines have been proposed as an attractive strategy to block bacteria or viruses at their entry points (5). Therefore, next-generation vaccines should ideally be mucosal vaccines in single, non-reactogenic, stable, and low efficacious doses that can elicit strong and robust humoral and acquired immunity against infectious pathogens (6). However, systemic administration of the naked DNA vaccine has been found to elicit low immunogenicity, and it is also rapidly degraded by the recipient’s cellular nuclease (7). Under such circumstances, a live attenuated bacterial vector (LABV) can be considered a potential delivery vector for delivering the DNA vaccine to the recipients’ cells safely.

In the 1990s, DNA and RNA-based vaccines became the fastest-growing vaccine technology, although the poor immune efficacy of plasmid DNA vaccination, as observed in clinical trials, became one of the major hurdles preventing its widespread use (8). Tang et al. (9) demonstrated that the direct injection of DNA-gold-coated microprojectiles elicited an immune response in mice models. However, despite developments and advancements in delivery approaches (e.g., gene guns and electroporation), the transfer efficiency of DNA vaccines has remained low, possibly due to its degradation by the recipient’s enzymes and nucleases (10). Notably, a high amount of purified naked DNA plasmid is required to trigger an immune response in larger animal models (11). Moreover, the lack of safe and effective adjuvants to boost the DNA vaccine-elicited immune response and insufficient knowledge of immune protection mechanisms pertaining to the DNA vaccine are some of the most significant hurdles to developing an effective DNA vaccine for infectious diseases (12).

In this context, LABVs, which have the ability to carry plasmid DNA as well as colonise the mucosal surface of recipient cells, have been proposed as a DNA vaccine carrier. The components of the outer membrane of LABV are highly immunogenic and can act as an adjuvant to enhance antigen-specific immunity through the expression of heterologous proteins, while also granting cross-protection against the bacterial vector itself (13). In addition, bacterial-vectored DNA vaccines are capable of eliciting robust mucosal and cellular immune responses in recipients because they have the ability to directly present the foreign antigen to the professional antigen-presenting cells (APCs) residing in mucosa-associated lymphoid tissue (14). Apart from this, these vaccines can be delivered through non-parenteral routes, such as intranasal and oral routes, thus offering a syringe-free and needle-free administration platform. As a result, it can be considered a good candidate for mass vaccination, especially during pandemics. Moreover, a relatively low cost is involved in developing and manufacturing recombinant bacterial-vectored DNA vaccines in large quantities (15). In addition, it does not require a biosafety level-3 (BSL-3) facility, and its disposal can be carried out in a relatively safe and easy manner, such as through autoclaving.

The first trial of DNA vaccine delivery using the attenuated invasive bacteria *Shigella* sp. was conducted in the 1990s (16), in which immunised mice models were observed to successfully develop the anti-β-galactoside specific antibody. Subsequently, the list of potential DNA vaccine carriers was extended to include other invasive and non-invasive LABVs, such as *Salmonella* sp., *Listeria* sp., *Vibrio* sp., and probiotic bacteria.

## Strategies for Developing LABV DNA Vaccines

**Figure 1:**
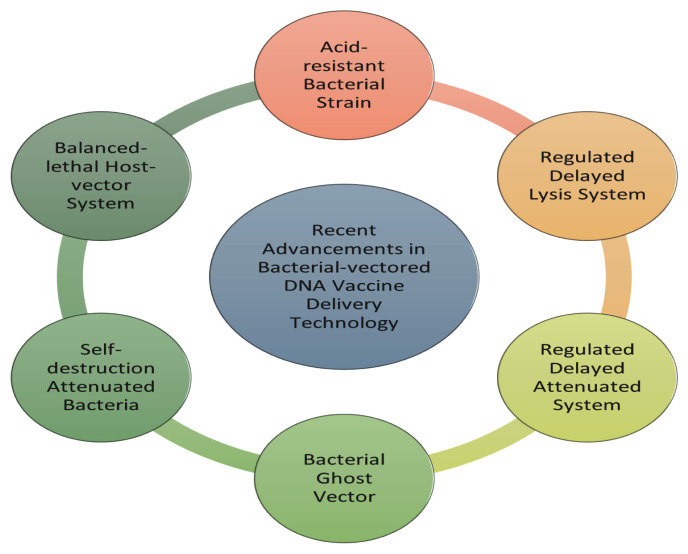
Recent advancements in bacterial-vectored DNA vaccine delivery technology

In the past two decades, researchers have made numerous efforts to develop and advance the technology related to the bacterial vector-based vaccine platform. Although most LABVs have been constructed through the mutation of their survival- and pathogenesis-related genes, they can still revert to their virulent state. Therefore, researchers have developed novel technologies to ensure LABVs remain in their weakened form and to further enhance their transfer efficiency, as shown in Figure 1.

### Balanced-lethal Host-vector System

Housekeeping genes are essential for regulating and maintaining the metabolism of bacteria, while also helping them adapt to different environments (17). Deletion of any essential gene of pathogenic bacteria makes them weak and unable to cause disease. Therefore, to maintain the viability of LABVs, essential nutrients need to be supplied to them from the external environment. To fulfil this need, the designed plasmid must carry the deleted essential gene to complement the mutated essential gene from the host’s chromosome. In this regard, Rui et al. (18) and Kang et al. (19) developed the *Asd*^−^ host/*Asd*^+^ plasmid vector for *Shigella flexneri* and *Salmonella tyhimurium*, respectively. The deletion mutation of the aspartate β-semialdehyde dehydrogenase (*asd*) gene in the host chromosome disabled the biosynthesis of diaminopimelate (DAP), which is necessary for bacterial cell wall synthesis (20). Consequently, the *asd*-mutated bacterial strains were unable to grow in the Luria-Bertani (LB) broth culture medium without the DAP supplement. As a result, the balanced-lethal host-vector system allowed the mutants to uptake and retain the recombinant plasmid carrying the essential gene in a relatively stable manner, ensuring its survival. Another study by Kim et al. (21) reported the development of a novel *GlmS*-based host-vector system for *Escherichia coli* and *S. typhimurium*. Both balanced-lethal host-vector systems demonstrated that *Asd*^−^ and *GlmS*^−^ mutants can survive in animal tissue, even in the case of insufficient or lack of required nutrients. Moreover, the balanced-lethal host-vector system enabled the elimination of the antibiotic selection system for plasmid maintenance.

### Self-destruction Attenuated Bacteria

The suicidal bacterial strain refers to bacteria that undergo autolysis after delivering the plasmid vector or foreign protein into the cytoplasm of the recipient. Notably, a selfdestructing *Listeria monocytogenes* strain was first described by Dietrich et al. (22). A *Listeria*-specific cell wall lysin derived from bacteriophage, *ply118* gene, and *hol118* gene can be cloned into the plasmid under the control of a *Listeria* constitutive intracellular activated promoter, P*_actA_* or P*_hly_* (22–24). After recombinant *L. monocytogenes* enter the host cytosol, the *actA* promoter triggers the synthesis of lysin or holin protein, causing the rupture of the cell wall of *L. monocytogenes*. Subsequently, the antigen-harbouring plasmid is released into the cytosol of infected macrophages (22, 23), following which the eukaryotic promoter P*_cmv_* drives the expression of the protein in the host cells. This confirms that the combination of dual-promoter shuttle plasmids can lead to the successful expression of foreign proteins in both *L. monocytogenes* and mammalian cells (22, 23).

### Bacterial Ghost Vector

Bacterial ghost vectors (BGs) are gram-negative bacteria composed solely of cell envelopes without any cytoplasmic content, although their cell surface structures remain intact. BGs can be produced using the cloned lysis gene *E* of *E. coli* bacteriophage ϕX174 in an inducible and repressible lactose operon and repressor system. In the presence of an inducer, gene *E* is activated, forming tiny lysis tunnels on the cell wall of the bacteria, which consequently leads to the release of its cytoplasmic content into the external environment (25). In previous studies, *Vibrio cholerae* BGs have been used as the vaccine delivery vector because they express heterologous proteins, such as reverse transcriptase of HIV and intimin protein of *Chlamydia* sp. (25, 26). This technology was later extended to include gram-positive bacteria, such as *Lactobacillus* sp. Furthermore, Hou et al. (27) successfully developed *Lactobacillus casei* ghosts using the holin gene derived from the bacteriophage of *L. casei* ATCC 393. In particular, the relatively large interior space of BGs makes them a suitable delivery vector for macromolecules, such as DNA vaccines and drugs.

### Regulated Delayed Attenuation System

Regulated delayed attenuation enables bacterial strains to retain their virulent form *in vitro*, allowing them to colonise the lymphoid tissues of the host effectively during the early immunisation period, which is followed by attenuation *in vivo*, thus avoiding disease (28). These bacterial strains commonly carry the mutated ferric uptake encoding gene *fur*, which plays a critical role in the iron uptake of pathogenic bacteria. Notably, *fur* family-related genes are responsible for the biosynthesis of virulence factors (29). In this context, the construction of regulated delayed attenuated *Salmonella* sp. and *Yersinia* sp. have been accomplished by replacing the constitutive promoter of acid resistance or shock regulatory genes, such as *RpoS*, *Fur*, *PhoPQ*, and *OmpR*, with the arabinose-inducible tightly regulated promoter *araC* P_BAD_ (30–33). Notably, an arabinose-regulated promoter is an apt choice for controlling the expression of virulent genes because it can switch the genes on or off based on the presence of arabinose. Overall, the regulated delayed attenuation system demonstrates good colonisation of attenuated bacteria during the initial immunisation period, thus facilitating the delivery of the DNA vaccine. Moreover, compared to the irregular survivability of the wild-type *Salmonella* strain, the regulated delayed attenuated *Salmonella* sp. gradually decreases in number over time (31).

### Regulated Delayed Lysis System

A regulated delayed lysis attenuated *Salmonella* strain (RASV) was first constructed and described by Kong et al. (34). Notably, a regulated delayed lysis system ensures autolysis of the attenuated bacterial strain after colonising and delivering the DNA vaccine to the host lymphoid tissue. Attenuation can be accomplished by deletion mutation of the *asdA* gene and by arabinose-regulated expression of the conditional lethal muramic acid encoding gene *murA*, which is crucial for bacterial cell wall synthesis (35, 36). In addition, an arabinose-regulated phage repressor gene, *C2*-derived bacteriophage P22, can be chromosomally inserted into the RASV to repress the transcription of genes under the control of the P22 P_R_ promoter. In the absence of arabinose, the *C2*-regulated promoter P22 P_R_ is activated, producing anti-sense mRNA that prevents the synthesis of any residual *asdA* and *murA* mRNA (36, 37). The reduction of these gene products eventually causes bacterial cell lysis. In a recent study, recombinant RASV was found to survive and colonise only in deep lymphoid tissues and to successfully deliver the DNA vaccine in the presence of arabinose (37). Furthermore, the deletion of the periplasm endonuclease I enzyme encoding gene *endA* has been found to enhance the survival rate of the plasmid upon its release into host cells (34).

### Acid-resistant Bacterial Strain

Acid tolerability is one of the most important criteria of LABVs that enables them to withstand a low-pH stomach environment and subsequently colonise the host’s gastrointestinal tract (GI) to deliver a DNA vaccine (38). Mutations in certain virulence-related genes, such as *rpoS*, *phoPQ*, and *fur* of LABVs, make them more acid-sensitive compared to their parent strains (39, 40). Improvement in the acid-resistant capability of LABVs ensures that a larger number of bacterial cells reach the GI, leading to the requirement for a low colony forming unit (CFU)/dose and fewer doses to elicit long-lasting and high-level protection against infectious diseases (41). In this context, previous studies incorporated the glutamate/arginine-dependent acid resistance gene *gad / AdiA-AdiC* into the multi-copy plasmid and chromosome of attenuated *Salmonella* strain under the control of a tightly regulated arabinose promoter (P_araBAD_) or rhamnose promoter (P_rhaBAD_) (40, 41). Both systems exhibited improved survival rates of the acid-sensitive attenuated *Salmonella* strain at pH 2.5, leading to an increased number of viable cells colonising in the lymphoid tissues of mice.

## Design of the Shuttle Plasmid

**Figure 2:**
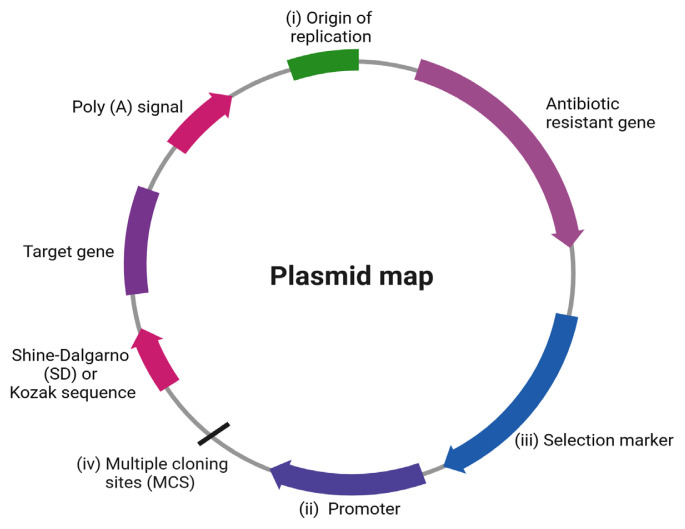
Essential components of a shuttle plasmid

A shuttle plasmid vector contains the desired antigens of bacteria or viruses encoding genes, which are later carried by LABVs. Notably, the delivery of the shuttle plasmid DNA to the host immune cell and the expression of heterologous proteins, either in bacterial delivery vehicles, mammalian cells or both, are integral to eliciting mucosal, humoral, and cell-mediated immune responses. A plasmid is commonly composed of several essential elements: i) the origin of replication *ori*; ii) promoters; iii) selection markers; and iv) multiple cloning sites (MCSs), as shown in Figure 2.

*Ori* refers to the specific site in the DNA sequence that initiates the replication of plasmid or bacterial genomes into billions of copies. Plasmids with a high copy number *ori* (500–700 copies/cell), such as ColE1 and pUC-*ori*, are widely used to construct cloning and expression plasmid vectors. However, the leaky problem associated with high copy number plasmids causes overproduction of the toxic gene product, which is detrimental to LABVs (42). Therefore, plasmids with a low copy number are preferable, since they allow only a few copies (10–15 copies/cell) of the plasmid to be produced per cell, thus limiting gene expression and preventing the accumulation of cloned toxic products, which eventually reduces the metabolic burden of recombinant LABVs (42).

Promoters are short DNA sequences usually found upstream of the genes that drive gene expression. A constitutive promoter is an unregulated promoter that allows for the transcription of genes under all circumstances *in vivo* (43). This means that using a constitutive promoter (e.g., P_sppA_, P_bla_, and P_rrnB P1_) allows toxic products to be produced constitutively, which might lead to the formation of a metabolic burden or could even be lethal to the bacteria (44). Hence, the ideal promoter for recombinant LABVs or LABs should be tightly regulated and inducible (43). For example, arabinose (P_araBAD_), rhamnose-inducible promoters (P_rhaBAD_), and T7*lac* promoters have often been employed to control the expression of toxic foreign genes, thereby minimising the metabolic burden and suppressing the mutated virulence genes of bacteria to prevent them from reverting to their virulent state (45, 46). Furthermore, eukaryotic promoters (e.g., P*_cmv_*, P*_rsv_*, and P*_EF1a_*) are known to drive the expression and production of heterologous proteins not only in mammalian cells but also in certain gram-negative bacteria (47). Recently, the dual-promoter shuttle plasmid vector, comprising both prokaryotic and eukaryotic promoters, has been observed to enable the expression of heterologous protein in both the bacterial vector and mammalian cells, thus further enhancing the immune response (48).

The selection markers enable the screening of those LABVs that successfully uptake the shuttle plasmid. Notably, antibiotic encoding genes, such as ampicillin (*bla*), chloramphenicol (*cmlA*), and kanamycin resistance genes (*aphA*), are some commonly used selection markers that confer antibiotic resistance abilities to recombinant bacteria, enabling them to grow in an antibiotic-selective environment (49). However, the transferability of the antibiotic resistance gene originating from DNA vaccines to the recipient’s gut microbiome poses significant safety concerns (50). In particular, it is advisable to avoid incorporating the ampicillin resistance gene into a DNA vaccine design due to the potential hypersensitivity of some patients to β-lactam antibiotics (51). Overall, an antibiotic-free (AF) shuttle plasmid is an important element of DNA vaccine development.

The fluorescence-based visual selection system became the first AF shuttle plasmid, as described by Solaiman and Somkuti (52), who employed a green fluorescent protein-encoding gene derived from jellyfish that enabled recombinant bacteria to fluoresce under UV illumination. It has also been reported that the overexpression of the host essential gene *FabI* allows for the selection of recombinant bacteria in the presence of chemical inhibitors, such as triclosan (49, 53). In this context, toxin-antidote (TA) systems, such as *hok/sok*, are widely studied alternatives that can be employed for the selection of recombinant bacteria. Such a system causes the recombinant bacteria to simultaneously produce a stable toxin (Hok) and an unstable anti-toxin (Sok) (54). As a result, a TA system is known to be responsible for bacterial plasmid maintenance (55). Notably, an RNA-based selection marker constructed using the 150 bp antisense-RNA regulator RNA-OUT was found to successfully repress the expression of a chromosomally integrated counter-selectable *sacB* levansucrase-encoding gene under the control of the RNA-IN promoter, enabling the growth of transformants in the presence of sucrose (56).

## Chemical and Genetic Adjuvants to Improve the Vaccine Efficiency

**Figure 3:**
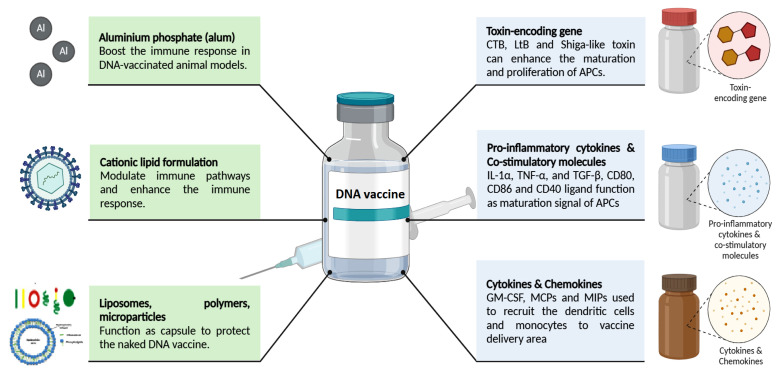
Adjuvants used to improve the efficiency of a DNA vaccine

The antigens encoding genes chosen as DNA vaccines are usually outer surface proteins or secretable toxins known to elicit the production of neutralising antibodies and are recognisable by immune cells (3). In particular, DNA vaccines are known to provide robust cellular and humoral immunity. However, low immunogenicity in larger animals and humans has remained a major hurdle to their widespread use. In this context, chemical or genetic adjuvants can be used in DNA vaccines to enhance the recipient’s immune response, as shown in Figure 3.

Notably, the chemical adjuvants commonly used by conventional vaccination platforms do not show their “depot effect” in DNA vaccines. This is because DNA vaccines only comprise antigen-encoding genes, which exhibit no direct interaction with chemical adjuvants when co-administered with them (57). Notably, aluminium phosphate (alum) is a well-known licensed adjuvant used in other vaccine platforms. Studies have shown that when used as an adjuvant in DNA vaccines, alum has the potential to enhance the immune response in animal models (58). Furthermore, Vaxfectin, a cationic lipid formulation, has been developed as a chemical adjuvant for DNA vaccines. This cationic lipid can modulate immune pathways, thereby enhancing the humoral-mediated immune response (59). Apart from this, other chemical adjuvants, such as liposomes, polymers, and microparticles, function as capsules to protect DNA vaccines from degradation, improve their expression and further enhance antigen-specific immune responses in animal models (60).

Genetic adjuvants, such as cytokine- or toxin-encoding genes, can be delivered using the same or different shuttle vectors. Therefore, genetic adjuvants can be expressed simultaneously with antigens. The most commonly used genetic adjuvants, such as cholera toxin B (CTB), heat-labile toxin (LtB), and Shiga-like toxin, are mucosal adjuvants, which contribute to enhancing the maturation and proliferation of professional APCs (61). Among these, CTB and LtB have been used both in animal models and clinical studies (62). However, a recent study found that although LtB enhances mucosal immunity, it leads to severe gastric inflammation and injury in animal models (63). Furthermore, pro-inflammatory cytokines (IL-1α, TNF-α, and TGF-β) and co-stimulatory molecules (CD80, CD86, and CD40 ligand), which act as maturation signals for APCs, can be incorporated into the DNA vaccine to increase the possibility of the APCs recognising and presenting the expressed antigens, subsequently enhancing adaptive immunity (64, 65). Cytokines and chemokines (GM-CSF, MCPs, and MIPs) have also been investigated with regard to their potential for recruiting blood-borne dendritic cells (DCs) and monocytes to the interstitial region of the vaccine delivery area (66). In recent years, DC-targeting peptides have emerged as a popular alternative, considering that they can direct the expressed antigen to the DC, facilitate the activation and maturation of DC and promote the differentiation of B and T cells (6, 67).

## LABV Candidates

**Figure 4:**
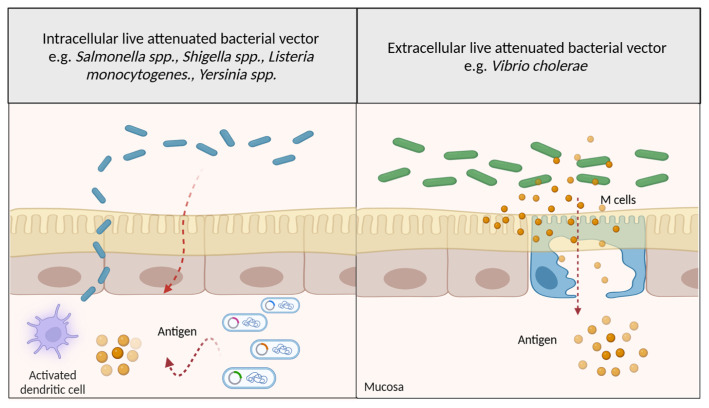
DNA vaccine delivery mechanisms of LABVs

The most commonly used LABVs employed to deliver DNA vaccines are enteropathogens, which can colonise the host’s intestine or penetrate their epithelial cells to deliver either plasmid DNA or the expressed foreign antigen. Subsequently, the foreign components are processed and presented to the APCs, thereby eliciting an immune response, as shown in Figure 4.

### Salmonella spp

In the 1990s, *Salmonella* strains, such as *S. typhimurium* and *S. typhi*, became the first strains used as DNA delivery vectors. They are considered good DNA delivery vectors because of their ability to survive under the stress of GI and invade gut-associated lymphoid tissues (GALTs), in turn eliciting the production of mucosal and cellular immunity (37). In addition, their outer membrane proteins, such as flagellin, can act as adjuvants to enhance the level of antigen-specific immunity (37).

Recombinant *Salmonella* strains (RASV) have been proven to express both foreign bacterial and viral antigens. Previous studies have shown that antigens of *S. pneumoniae* and *S. aureus* can be successfully expressed by RASV, while antigen-specific IgG and IgA levels were detected in sera, vaginal lavage, and faecal extracts in mice model. Furthermore, the recombinant RASV strains expressing antigens of *S. pneumonia* were observed to induce a balance Th1/Th2 immunity response, while the ones expressing antigens of *S. aureus* were shown to develop Th2-biased immunity response (1, 19, 34). RASV has also been proposed as a delivery vehicle for viral antigens, such as the hemagglutinin (HA) gene of the influenza virus, the nucleocapsid protein of the foot-and-mouth disease virus and the spike protein of SARS-CoV-2. Furthermore, Kong et al. (2) demonstrated that rRASV has the ability to elicit HA-specific IgG in mice model. In contrast, in a study conducted by Van et al. (68), an rRASV carrying the same HA gene was unable to elicit HA-specific IgG and increase the IL-4 and IFN-γ levels in immunised mice model. Furthermore, RASV carrying the plasmid encoding the spike protein of SARS-CoV-2 (S) and a multi-epitope vaccine construct (RBD-HR-N-RdRp) was able to elicit sera S-specific IgG and cellular immune responses in a mice model (69, 70).

### Listeria monocytogenes

*Listeria monocytogenes* are intracellular anaerobes whose intracellular life cycle enables them to invade, survive, and replicate in non-phagocytic and phagocytic cells. Therefore, this bacterium has been proposed as a potential DNA vaccine carrier for transferring plasmid DNA into the host cytoplasm to drive the expression of the heterologous protein (23). Studies have shown that the recombinant *L. monocytogenes* strain (Lmdd-gag) is able to elicit HIV Gag-specific cell-mediated and mucosal immune responses in rhesus monkey groups (71). Furthermore, in a study by Johnson et al. (72), *L. monocytogenes* carrying the nucleoprotein (NP) of Influenza A-encoded plasmid successfully elicited the production of NP-specific IFN-γ spot in a mice model, although no detectable NP-specific IgG and mucosal IgA were observed in human volunteers. Recently, Pownall et al. (73) developed a novel triple mutation of the *L. monocytogenes* strain (Lm3Dx) that successfully delivered the surface antigen 1 of *Neospora caninum* (NcSAG1), eliciting cellular-mediated (IFN-γ and IL-5) and humoral immunity in a mice model.

### Shigella spp

The unique features of *Shigella* spp. make them attractive vaccine delivery vectors. These features include their ability to escape from endosomes to enter the host cytoplasm and their natural target lymphoid tissue in the mucosa of the intestine, which has been found to elicit immunity without causing severe pathology or disease (74). In particular, *S. flexneri* 2a, along with the deletion mutation of the *asdA* gene, is a common strain used as a vaccine delivery carrier (16). Unlike *Salmonella* spp., this strain can be restricted to infecting only the digestive system without spreading to the bloodstream. Furthermore, the recombinant *S. flexneri* 2a CVD1204 strain elicited the production of heat-labile enterotoxin (LTh) of *E. coli* specific-IgA and IgG in 40% of immunised mice models (75). In a study by Zhang et al. (76) that employed a recombinant *S. flexneri* SH02 vaccine candidate, oral-priming subcutaneous-boosting (heterologous vaccination group) immunised mice developed higher levels of urease B-heat shock protein A (UreB-HspA)-specific sera IgG and sIgA compared to the homologous oral-priming group. UreB-HspA-specific IFN-γ and IL-17A-secreting CD154+ T cells were produced in both the homologous and heterologous vaccination groups. Unfortunately, there is a lack of suitable animal models for evaluating the efficacy of recombinant *Shigella* spp., since they are host-restricted. Therefore, developing a suitable small animal model for this purpose is critical (77).

### Vibrio cholerae

CTB of *V. cholerae* functions as a suitable adjuvant for enhancing the immune response in animal models, with its large genomic capacity enabling the insertion of a greater number of heterologous genes, making it a potential vaccine carrier. In a previous study, two doses of recombinant *V. cholerae* expressing initimin (EaeA) of enterohemorrhagic *E. coli* were able to elicit the production of sera anti-EaeA IgG and non-detectable anti-EaeA-IgA in a mice model (78). In a similar study, orally administered EaeA expressing *V. cholerae* CVD-103-HgR successfully elicited the production of sera EaeA-IgA in rabbit models (79).

### Yersinia spp

*Yersinia* spp. persists in host tissues for several days, while their lipopolysaccharide O chain can act as an adjuvant to boost humoral-mediated immunity, indicating that they can be used as potential DNA vaccine delivery vehicles (80). In this context, Al-Mariri et al. (80) constructed an attenuated *Y. enterocolitica* serotype O9 by removing its virulence plasmid (pYV) to ensure that it can be directed towards and enter host APCs. Notably, attenuated *Y. enterocolitica* carrying bacterioferritin (BFR) and the P39 antigen of *Brucella abortus* encoding plasmid has been found to induce antigen-specific IgG1 and IgG2a, as well as Th1-type immune responses, in intragastric immunised mice models (80). In another study, recombinant attenuated *Y. pseudotuberculosis*, which was employed to deliver the V-antigen-encoding plasmid (LcrV) of *Y. pestis*, successfully induced balanced Th1/Th2 responses and the production of CD4+ and CD8+ cells secreting IL-2, IL-17A, and TNF- α (33, 81).

## Conclusion

In recent years, the emergence of drug-resistant bacteria and the COVID-19 pandemic have imposed a huge burden on the global healthcare system. In this context, vaccination has emerged as the best prophylactic method for combating the spread of infectious pathogens. However, most currently-licensed vaccines are inactivated vaccines, adenovirus-vectored vaccines, or mRNA vaccines, which require a cold chain for storage and transportation. This requirement greatly increases the cost of vaccines, leading to relatively low vaccination rates in undeveloped and developing countries. In such a scenario, a cold-chain-free single-dose vaccine is the best alternative. DNA vaccines have emerged as the most attractive platform for this purpose, owing to their fast development, safety, and cost effectiveness. However, the low transfection efficiency of DNA vaccines in larger animals and humans has limited their usage. The use of LABV as a DNA vaccine delivery vehicle overcomes this problem while also ensuring a robust immune response in the recipient. Therefore, be it live attenuated bacteria or LABV, bacteria continue to be an important player in our vaccine development toolbox.

## Abbreviations

DNA: Deoxyribonucleic acid
LABV: Live attenuated bacterial vector
MDR: Multi-drug resistant
BGs: Bacterial ghost vector
GM-CSF: Granulocyte-macrophage colony-stimulating factor
MCPs: Monocyte chemoattractant proteins
MIPs: Macrophage inflammatory proteins
RASV: Recombinant attenuated *Salmonella* vector
